# Coronary artery aneurysm formation after paclitaxel-coated balloon-only intervention for *de novo* coronary chronic total occlusion

**DOI:** 10.3389/fcvm.2022.1039316

**Published:** 2023-01-04

**Authors:** Eun Jung Jun, Eun-Seok Shin, Bitna Kim, Eu-Vin Teoh, Chong-Mow Chu, Sunwon Kim, Houng Bang Liew

**Affiliations:** ^1^Department of Cardiology, Ulsan University Hospital, University of Ulsan College of Medicine, Ulsan, South Korea; ^2^Cardiology Department and Clinical Research Center, Queen Elizabeth Hospital II, Kota Kinabalu, Malaysia; ^3^Department of Cardiology, Korea University Ansan Hospital, Ansan-si, South Korea

**Keywords:** coronary artery aneurysm, paclitaxel-coated balloon, drug-coated balloon, *de novo* coronary artery, chronic total occlusion

## Abstract

**Background:**

Although coronary artery aneurysm (CAA) is an uncommon complication of drug-coated balloon (DCB) treatment, the incidence and mechanisms CAA formation after DCB intervention for chronic total occlusion (CTO) remains to be clarified. The aim of this study was to investigate the incidence of CAA after DCB intervention for the treatment of CTO of coronary arteries.

**Materials and methods:**

This was a retrospective analysis of 82 patients, contributing 88 vessels, who underwent successful DCB-only treatment for *de novo* CTO lesions. Follow-up angiography was performed in all cases, at a mean 208.5 (interquartile range [IQR]: 174.8 to 337.5) days after the index procedure.

**Results:**

CAA was identified in seven vessels, in seven patients, at the site of previous successful DCB-only treatment. Of these, six were fusiform in shape and one saccular, with a mean diameter of 4.2 ± 1.0 mm and length of 6.7 ± 2.6 mm. Six CAAs developed at the CTO inlet site, and all CAAs occurred at the lesions following dissection immediately after DCB treatment. CAAs were not associated with an increased risk of major clinical events over the median follow-up of 676.5 (IQR: 393.8 to 1,304.8) days.

**Conclusion:**

The incidence of CAA after DCB-only treatment for CTO lesions was 8.0% in this study. Further research is warranted, using intravascular imaging, to clarify the mechanism of DCB-related CAA formation and prognosis.

## Introduction

A coronary artery aneurysms (CAAs), defined as an abnormal coronary dilation exceeding the diameter of the adjacent normal segments of an artery by at least 50%, are not common occurrences (1, 2). The incidence of CAAs after percutaneous coronary intervention (PCI) is low, with an estimated rate of 3.9% after percutaneous transluminal coronary angioplasty (PTCA) (3) and 0.7 to 3.4% after implantation of drug-eluting stent (DES) (4). With balloon angioplasty, arterial dissection, and disruption of the tunica media of the arterial wall are considered as possible causes of CAA formation. However, the underlying mechanisms of CAA formation after drug-coated balloon (DCB) treatment have not been fully established.

Paclitaxel is a highly hydrophobic agent that allows the drug to substantially invade the vascular wall of the artery and inhibits smooth muscle cell proliferation by modulating microtubule formation and upregulating proapoptotic factors (5, 6), resulting in vessel enlargement (positive remodeling) after PCI. However, with proapoptotic effects, there is the possibility for wall thinning and aneurysm formation (7). Although chronic total occlusion (CTO) lesions implanted with DES appear to increase the risk of CAA formation (8), CAA formation after DCB intervention in CTO has not been previously reported. Therefore, our aim was to investigate the incidence of CAA after DCB intervention in patients with CTO lesions using an international multicenter DCB registry.

## Materials and methods

### Study population

From the registry, we identified 82 patients contributing 88 vessels with *de novo* CTO lesions, successfully revascularized using DCB-only, and underwent follow-up coronary angiography, between 15 April 2015 and 25 October 2019. Procedures were performed by operators experienced in treating *de novo* CTOs with DCB, at four teaching hospitals in Malaysia and South Korea (Queen Elizabeth Hospital II, Ulsan University Hospital, Ulsan Medical Center, and Korea University Hospital). Excluded were patients with DCB treatment for CTO due to occlusive in-stent restenosis, additional stents placed in the target vessel during the index procedure, or unstable hemodynamic conditions at presentation.

### Statement of ethics

The study protocol was approved by the institutional review board or ethics committee of each participating center and all patients provided written informed consent. The international multicenter DCB registry used, the Impact of Drug-coated Balloon Treatment in *de Novo* Coronary Lesion, is registered at Clinicaltrials. gov (NCT04619277).

### Procedures

All interventions were performed using an antegrade recanalization approach. Cardiac catheterization, premedication, and medication during the intervention were used according to standards of practice at each participating hospital and current guidelines. Angiography of the target vessel was performed at the following three time points: baseline, before the intervention; after successful guidewire passage and pre-dilation of the occluded lesion; and at the end of the intervention, following the treatment with DCB.

The intervention was performed according to the International DCB Consensus, Asia-Pacific Consensus, and provisional approach to DCB PCI (9–11). According to these recommendations, for *de novo* CTOs, balloon pre-dilation was performed, using a balloon-to-vessel ratio of 0.8–1.0, as recommended. For our study, pre-dilation was considered successful and suitable as a DCB-only strategy if the residual stenosis was not more than 30% of the vessel diameter on direct observation and a thrombolysis in myocardial infarction (TIMI) flow grade 3 was achieved. After pre-dilation balloon angioplasty, stenting was deferred in any types of dissections provided there was TIMI flow grade 3. This point was different from that recommended by the International DCB Consensus group. Therefore, following pre-dilation balloon angioplasty, stenting was not considered solely based the presence of major dissections (type C or higher), categorized according to the National Heart, Lung, and Blood Institute classification system (12). As per current guidelines, stent insertion without DCB is recommended for a TIMI flow grade < 3. The DCB was inflated to its nominal pressure for at least 60 s, being careful to extend the DCB at least 2 mm beyond the initial pre-dilatation. All the DCBs were coated with 3.0 μg/mm^2^ paclitaxel combined with either iopromide (SeQuent Please© by B. Braun, Germany) or urea (In. Pact Falcon© by Medtronic-Invatec, Italy) as a carrier for the drug. After PCI, even though the duration of the prescribed dual-antiplatelet therapy was left to the attending doctors’ discretion, almost all patients received dual-antiplatelet therapy for at least 1 month with the lifelong continuation of aspirin once daily afterward.

### Quantitative coronary analysis and clinical follow-up

Angiographic follow-up was performed in all 82 patients, at a median follow-up of 208.5 (interquartile range [IQR]: 174.8 to 337.5) days, with outcomes assessed using quantitative coronary analysis. Acute lumen gain or late lumen loss was calculated as the difference between the minimal lumen diameter after the procedure and at follow-up. CAAs were defined as focal dilation of at least 1.5 times the adjacent normal segments and divided into two types (saccular aneurysm: transverse > longitudinal diameter; fusiform aneurysm: transverse < longitudinal diameter) (1, 2, 13). All patients were clinically followed up to a median of 359 (IQR: 58.8 to 1027.0) days after follow-up coronary angiography to confirm the clinical impact of CAA. Follow-ups by telephone interviews and outpatient clinic visits were also considered in our study to assess the occurrence of major adverse cardiac events (MACE), defined as a composite of cardiac death, non-fatal myocardial infarction (MI), target vessel revascularization (TVR), and target vessel thrombosis.

### Statistical analysis

Categorical variables are presented as a count and relative frequency (percentage) and continuous variables as a mean (standard deviation) or median (first and third quartiles), as appropriate for the data distribution evaluated using the Kolmogorov–Smirnov test for normality. For demographic information, continuous data were summarized by descriptive statistics (number of cases, mean, and standard deviation) and categorical data by frequency and proportion. Between-group comparisons were evaluated using an independent two-sample *t*-test or Wilcoxon rank-sum test for continuous variables and a chi-squared test or Fisher’s exact test for categorical variables, as appropriate. All probability values were two-sided, with a *p*-value < 0.05 considered significant. *Post-hoc* analyses were performed to compare the two groups in each sub-types of dissection. Cochran–Armitage test for trend used to assess for the presence of an association between the severity of coronary dissection and proportion of CAA. All statistical analyses were performed using R (version 3.6.3; R Foundation for Statistical Computing, Vienna, Austria).

## Results

### Baseline characteristics

Patient baseline clinical characteristics are summarized in Table 1 according to the presence or absence of procedure-related CAA, namely the CAA and without CAA group, respectively. Overall, the mean age of the study group was 56.8 ± 11.1 years, with 80.5% being men and just over one third having diabetes mellitus. The mean left ventricular ejection fraction was 51.4 ± 12.4%. Other relevant characteristics included chronic stable angina in 58.5% of patients, acute coronary syndrome in 41.5%, and significant coronary artery disease in more than one main vessel in 52.4%. The two groups were comparable on all baseline characteristics, except for previous MI, which was higher for the CAA (57.1%) than without CAA (17.3%) group (*p* = 0.013).

**TABLE 1:** Baseline patient characteristics according to the presence of aneurysm after DCB-only treatment.

	CAA *N* = 7 patients	Without CAA *N* = 75 patients	*P*-value
Age, years	53.3 ± 7.3	57.1 ± 11.3	0.384
Male	7 (100)	59 (78.7)	0.173
Hypertension	5 (71.4)	51 (68.0)	0.852
Hypercholesterolemia	2 (28.6)	37 (55.2)	0.179
Diabetes	2 (28.6)	31 (41.3)	0.510
Current smoker	3 (42.9)	14 (18.9)	0.165
Previous myocardial infarction	4 (57.1)	13 (17.3)	0.013
Family history of coronary artery disease	3 (42.9)	22 (29.3)	0.457
Left ventricular ejection fraction, %	54.0 ± 15.4	51.2 ± 12.2	0.598
Clinical presentation			0.127
Chronic stable angina	6 (85.7)	42 (56.0)	
Acute coronary syndrome	1 (14.3)	33 (44.0)	

Values are mean ± SD or number (percentage). CAA, coronary artery aneurysm; DCB, drug-coated balloon.

### Angiographic and procedural characteristics

The angiographic and procedural data are presented in Table 2. Most vessels were treated *via* radial access. The median SYNTAX (SYNergy between PCI with TAXUS and cardiac surgery) score was 22.9 (IQR: 16.5 to 30.0), with the distribution of scores as follows: < 23, 50.6%; 23–32, 30.1%; and ≥ 32, 19.3%. The left anterior descending artery was treated in 52.3% of cases, with a higher prevalence in the CAA (85.7%) than the without CAA (49.4%) group (*p* < 0.001). The use of scoring balloons and the mean number of DCBs used were similar between the two groups. However, the mean pre-dilation balloon diameter was significantly higher in the CAA than in the without CAA group (2.8 ± 0.6 vs. 2.3 ± 0.5 mm, respectively, *p* = 0.008). The diameter of the DCB used was significantly larger in the CAA than in the without CAA group (3.0 ± 0.4 vs. 2.6 ± 0.4 mm, respectively, *p* = 0.028). The pre-dilation balloon and DCB-to-reference vessel ratio, however, were not different between the two groups. The mean DCB length and inflation time were also comparable between the two groups.

**TABLE 2:** Angiographic and procedural characteristics according to the presence of aneurysm after DCB-only treatment.

	CAA *N* = 7 vessels	Without CAA *N* = 81 vessels	*P*-value
Radial artery access	5 (71.4)	65 (81.3)	0.530
Total syntax score	24.6 ± 8.6	22.8 ± 9.4	0.612
Number of diseased vessels	1.7 ± 0.8	1.9 ± 0.9	0.658
Targeted vessel			0.131
Left anterior descending artery	6 (85.7)	40 (49.4)	
Left circumflex artery	0	28 (34.6)	
Right coronary artery	1 (14.3)	13 (16.0)	
Scoring balloon used	1 (14.3)	13 (19.4)	0.742
Predilation balloon diameter, mm	2.8 ± 0.6	2.3 ± 0.5	0.008
Predilation balloon to reference vessel ratio	1.0 ± 0.2	0.9 ± 0.2	0.074
Predilation balloon maximal pressure, atm	12.6 ± 1.9	13.7 ± 4.0	0.203
Number of DCB used	1.6 ± 0.5	1.5 ± 0.6	0.913
DCB diameter, mm	3.0 ± 0.4	2.6 ± 0.4	0.028
DCB to reference vessel ratio	1.0 ± 0.1	0.9 ± 0.2	0.162
DCB length, mm	45.7 ± 21.5	38.3 ± 16.1	0.259
DCB maximal pressure, atm	7.4 ± 1.3	9.4 ± 2.8	0.004
DCB inflation time, second	87.3 ± 69.7	75.5 ± 24.6	0.673
Dissection after DCB			< 0.001
None	0	41 (50.6)	< 0.001
A	0	12 (14.8)	< 0.001
B	3 (42.9)	23 (28.4)	< 0.001
C	4 (57.1)	5 (6.2)	< 0.001

Values are mean ± SD or number (percentage). CAA, coronary artery aneurysm; DCB, drug-coated balloon.

**FIGURE 1:**
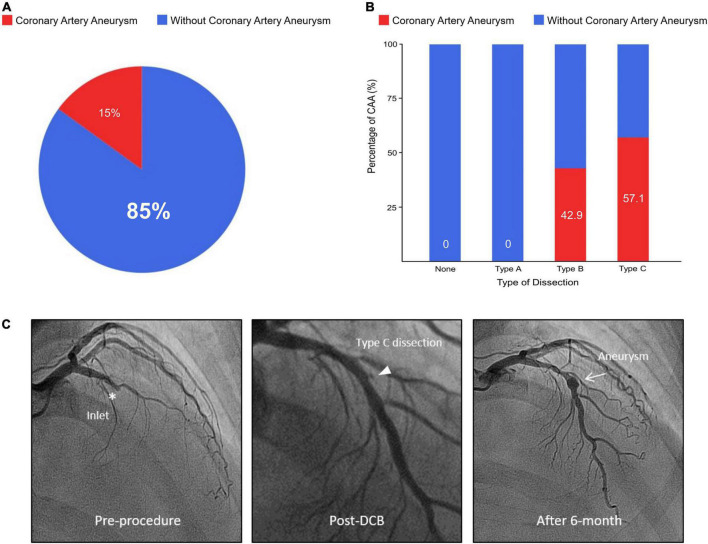
Percentage of coronary artery aneurysm according to dissection type after DCB treatment and representative case. **(A)** Proportion of aneurysm in dissection following DCB treatment. **(B)** Relationship between dissection type and percentage of CAA. **(C)** A representative case of 56-year-old male with stable angina. His only coronary risk factor was a family history of coronary artery disease. His coronary angiography shows a chronic total occlusion of left anterior descending artery. After successful pre-balloon angioplasty, the lesion was treated with a 3.5 × 20 mm DCB, and the DCB to reference vessel ratio was 1.2. After DCB treatment, there was a 17% residual diameter stenosis with normal antegrade flow and type C dissection at the inlet site of chronic total occlusion. Six-month later, follow-up angiography confirmed a fusiform aneurysm (5.1 × 6.5 mm) at the dissected lesion of inlet. He has been well without angina for 526-day. DCB, drug-coated balloon; CAA, coronary artery aneurysm.

Dissection after DCB treatment occurred in 47 vessels (53.4%), with the distribution as follows: type A, 12 vessels; type B, 26 vessels; and type C, 9 vessels (Table 2). A type C dissection was more prevalent in the CAA than in the without CAA group (57.1 vs. 6.2%, *p* < 0.001) (Figure 1). No aneurysm occurred in the absence of dissection or in the presence of dissection type A. In Cochran–Armitage test for trend, there was a significant association between the severity of coronary dissection and proportion of CAA, the more severe dissection, the higher CAA incidence (*p* < 0.001) (Supplementary Table 1).

**FIGURE 2:**
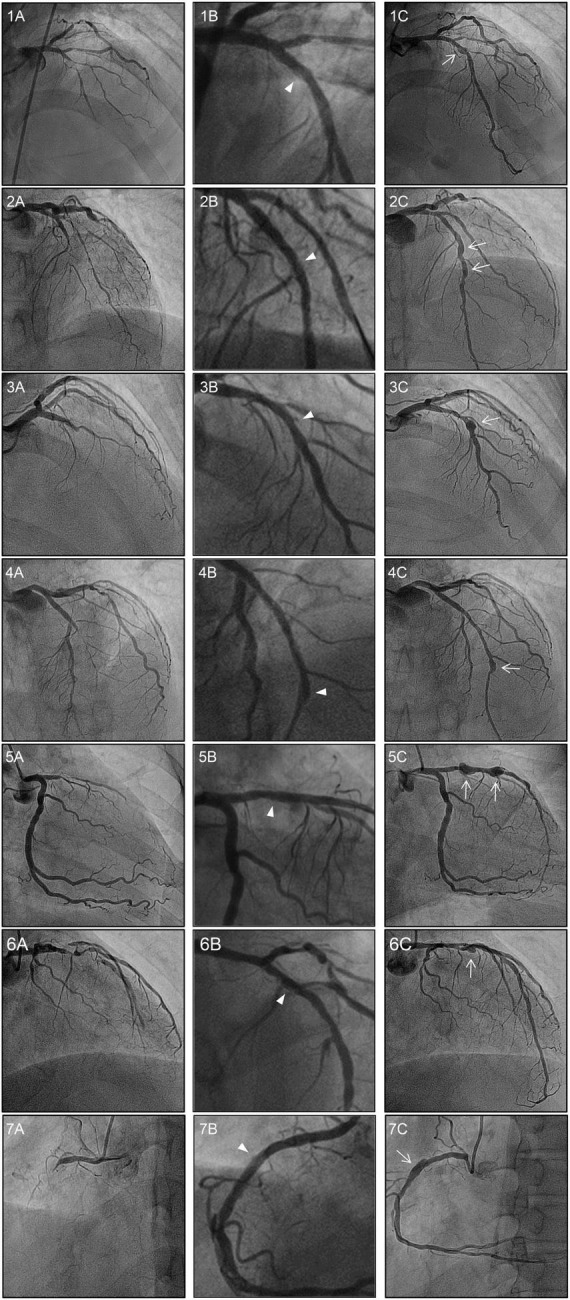
Baseline, post-DCB, and follow-up coronary angiograms for the 7 cases of aneurysm formation. Angiograms presented were obtained **(A)** pre-procedure, **(B)** post-DCB; and **(C)** at follow-up. The dissections and aneurysms are shown by the arrowheads and arrows, respectively. All vessels were treated with drug-coated balloon alone for CTO lesions. The aneurysm developed at the inlet of the target lesion in six cases, with the exception of case 4 in which the aneurysm developed at the outlet of the CTO lesion. Of note, for case 2, aneurysms developed at the inlet and the mid-segment site; while for case 5, aneurysms developed at the inlet and outlet sites of the CTO lesion. DCB, drug-coated balloon; CTO, chronic total occlusion.

Only 14.9% of dissections were associated with an aneurysm, with all 7 cases of CAA being associated with a dissection. Of these, there was one case of aneurysmal dilation immediately after the procedure, secondary to dissection. However, when this lesion was confirmed by follow-up angiography 1 year later, the dissection disappeared, but the aneurysm slightly increased. In the without CAA group, 3 cases of dissection, without aneurysm, were observed on follow-up coronary angiography, with the dissection resolving in 96.6% of these cases. Representative images are shown in Figure 2.

### Quantitative coronary angiography according to the presence or absence of aneurysm

Immediately after DCB treatment, there were comparable increases in the reference vessel diameter, minimal lumen diameter, and diameter stenosis in both groups (Table 3). In addition, the acute lumen gain was comparable in both groups (CAA group, 1.6 ± 0.4 mm; without CAA group, 1.6 ± 0.4 mm; *p* = 0.795). The minimal lumen diameter was maintained in both groups, with no between-group difference in the diameter stenosis on follow-up. The late lumen loss was also not significantly different between the two groups, at -0.28 ± 0.52 mm for the CAA group and 0.10 ± 0.56 mm for the without CAA group (*p* = 0.086).

**TABLE 3:** Quantitative coronary angiography according to the presence of aneurysm after DCB-only treatment.

	CAA *N* = 7 vessels	Without CAA *N* = 81 vessels	*P*-value
Post-DCB treatment			
Reference vessel diameter, mm	2.9 ± 0.2	2.9 ± 0.6	0.922
Lesion length, mm	45.5 ± 21.0	38.4 ± 16.0	0.279
Minimal lumen diameter, mm	1.6 ± 0.4	1.6 ± 0.4	0.838
Diameter stenosis,%	29.5 ± 8.6	30.8 ± 8.9	0.716
Acute lumen gain, mm	1.6 ± 0.4	1.6 ± 0.4	0.795
Follow-up			
Reference vessel diameter, mm	2.8 ± 0.3	2.9 ± 0.6	0.567
Lesion length, mm	45.7 ± 21.0	38.8 ± 16.2	0.295
Minimal lumen diameter, mm	1.9 ± 0.4	1.5 ± 0.7	0.176
Diameter stenosis,%	35.4 ± 9.5	38.6 ± 20.7	0.692
Late lumen loss, mm	−0.28 ± 0.52	0.10 ± 0.56	0.086
Follow-up duration, day, median (IQR)	155 (93–202)	220 (181–348)	

Values are mean ± SD or number (percentage). CAA, coronary artery aneurysm; DCB, drug-coated balloon.

### Aneurysm characteristics

The characteristics of the aneurysms are summarized in Table 4. Of the seven patients who developed CAA, six had stable angina and one acute coronary syndrome. Of the seven CAAs, six developed in the left anterior descending artery and one in the right coronary artery. All aneurysms occurred in men, with two aneurysms in one patient and another patient developing a diffused aneurysm. In all cases of CAA, vessel dissection occurred immediately after DCB treatment, including three type B dissections and four type C. Of the seven aneurysms, six were fusiform in shape, with the other being saccular in shape in a patient with acute coronary syndrome. Six aneurysms occurred at the inlet site of the CTO lesion and two at the outlet site. The average diameter of the aneurysm was 4.2 ± 1.0 mm, with an average length of 6.7 ± 2.6 mm.

**TABLE 4:** Procedure-related aneurysmal characteristics after DCB-only treatment.

	Baseline				Post-DCB				Follow-up					
	Clinical presentation	Vessel	Age	Sex	DCB to reference vessel ratio	Reference vessel size (mm)	Lesion length (mm)	NHLBI dissection type	Location	Types of aneurysm	Aneurysm transverse diameter (mm)	Longitudinal length (mm)	CAG follow-up duration (day)	MACE
1	Stable angina	LAD	57	Male	1.1	2.8	30.2	B	Inlet	Fusiform	4.0	4.5	155	None
2	Stable angina	LAD	53	Male	0.8	2.7	79.8	B	Inlet & Mid (diffuse)	Fusiform	3.0 & 2.8	4.3 & 4.2	202	None
3	Stable angina	LAD	56	Male	1.2	2.8	20.4	C	Inlet	Fusiform	5.1	6.5	182	None
4	Stable angina	LAD	61	Male	0.9	3.3	40.3	C	Outlet	Fusiform	3.5	6.3	367	None
5	Stable angina	LAD	38	Male	1.0	3.0	60.3	C	Inlet & Outlet	Fusiform	5.1 & 5.3	8.4 & 7.8	93	None
6	ACS	LAD	55	Male	1.1	2.7	30.3	B	Inlet	Saccular	4.1	5.8	134	None
7	Stable angina	RCA	53	Male	1.1	2.8	57.2	C	Inlet	Fusiform	4.6	12.3	72	None

ACS, acute coronary syndrome; LAD, left anterior descending artery; RCA, right coronary artery; DCB, drug-coated balloon; NHLBI, National Heart, Lung and Blood Institute classification system for coronary artery dissection types; CAG, coronary angiogram; MACE, major adverse cardiac event.

### Clinical outcomes

No aneurysm was associated with a MACE over the follow-up period. Moreover, there were no clinical events in the CAA group over the follow-up period (Supplementary Table 2). In the without CAA group, however, there were 2 occurrences of cardiac death, 1 occurrence of non-fatal MI, and 13 occurrences of TVR. There was no occurrence of target vessel thrombosis among patients treated with DCB alone.

## Discussion

The main findings of our study are as follows. First, the incidence rate of CAA after DCB-only intervention in patients with CTO lesions was 8.0%. Second, the more severe the dissection, the more likely an aneurysm is to form. However, the occurrence of a CAA did not increase the risk of MACE over the follow-up period.

The incidence rate of CAA after PTCA is approximately 5% (3, 13), increasing to 10% after directional coronary atherectomy (14). In non-CTO lesions, aneurysm rates of 1.3% after paclitaxel-coated DES, 0.8% after sirolimus-coated DES implantation (15), and 0.8% after paclitaxel-coated DCB treatment (7) have been reported. The 8.0% rate of aneurysms in our study, at a median follow-up of 208.5 days is higher than that of balloon angioplasty and approximately 10-fold greater than the rate for DES or DCB treatment in non-CTO lesions.

Drug administration by balloon causes much higher initial tissue concentrations of paclitaxel (300 μg/g tissue) than DES (3.2 ± 1.8 μg/g tissue) (16, 17). Although only part of the drug-coated balloon is delivered to the vessel, the use of a balloon might still promote additional pharmacological effects. While positive effects, such as reduction of neointimal hyperplasia and positive remodeling, result from an upregulation of proapoptotic factors (6), possible sequelae of the upregulation of apoptosis might be arterial wall thinning and aneurysm formation. CAA formation appears to be higher in complex lesions and after application of DES for CTO lesions (4, 8).

Although dissection is considered as one of the causes of CAA formation after PTCA, the mechanism of aneurysm formation after DCB treatment has not yet been identified. In our study, after DCB treatment, dissection was detected in 53.4% of all lesions treated and there was no aneurysm in the absence of dissection or in type A dissection. Noteworthy, all CAAs occurred at the dissection site, with more than half being associated with type C dissections. In fact, a type C dissection was only identified in 6.2% of lesions without aneurysm formation. Our findings indicate that the more severe the dissection, the more likely the occurrence of CAA. Of note, while CAAs occur at a site of dissection, not all dissections produce an aneurysm.

In our study group, the majority of CAAs were observed at the inlet site (85.7%) of the target lesions. In CTO lesions, wire penetration is very difficult at the inlet and, thus, multiple severe dissections are likely to occur after DCB treatment. Treatment of such lesions, resulting in medial or adventitial dissections, followed by DCB may increase the likelihood of aneurysm formation. Therefore, hybrid PCI, such as proximal spot stenting in the region of severe dissections of the inlet and DCB treatment in the distal lesion, could be helpful in preventing aneurysmal formation with CTO PCI.

Coronary angiography cannot provide further information for patients with mild ectasia or small aneurysms. This could lead to an underestimation of the actual size of the aneurysm or even overlooking a CAA that a thrombus or plaque may occlude. Coronary angiography is also limited in its ability to differentiate between a true aneurysm and pseudoaneurysm. IVUS provides transmural images of the coronary arteries, allowing information on the arterial wall structure and luminal composition to be derived. This is important for differentiating between types of aneurysms which may have very different prognostic outcomes.

In the present study, aneurysms were not associated with an increased risk of MACE. However, these results should be interpreted with caution considering the small size and potential for selection bias. Large cohort prospective studies with long-term follow-up are needed to confirm our results and to determine the true burden of CAAs as a complication of DCB treatment, to clarify the underlying pathophysiological mechanism of CAA formation, and, ultimately, to determine the long-term safety of DCB and to address the challenges of managing such complications.

Our study has several limitations. Foremost, this was a retrospective study and, although a large multicenter database was used, selection bias cannot be defined. The incidence rate of CAA after DCB treatment was 8.0%. However, this is not representative of the true occurrence of aneurysm formation after DCB due to the lack of comprehensive data and the specific time at which CAAs develop after DCB treatment, which is affected by various parameters, such as the time of angiographic investigation following DCB treatment and different inclusion criteria for further investigations after PCI. Lastly, CAA development was asymptomatic in all patients. Thus, the number of aneurysm formations among patients who refused follow-up angiography is unknown.

## Conclusion

Coronary artery aneurysm formation after DCB intervention for CTO lesions was identified in 7 of 88 cases of PCI with DCB, for an incidence rate of 8.0%. This rate is 10-fold higher than the rate for DES or DCB treatment for non-CTO lesions. CAA was not associated with an increased risk for MACE during follow-up. Results of our study should be interpreted with caution considering the small sample size and potential for selection bias. Further research is necessary regarding the mechanism of occurrence and prognosis of CAA related to DCB treatment using an intravascular imaging tool.

## Data availability statement

The original contributions presented in this study are included in the article/Supplementary material, further inquiries can be directed to the corresponding authors.

## Ethics statement

Written informed consent was obtained from the individual(s), and minor(s)’ legal guardian/next of kin, for the publication of any potentially identifiable images or data included in this article.

## Author contributions

E-SS contributed substantially to the design of the present study, had full access to the database, and take responsibility for the integrity and of the data and the data analyses. EJJ provided the first draft of the manuscript. EJJ and BK performed the data analyses. BK performed the statistical analyses. All authors participated in the interpretation of data, critically revised the manuscript, confirmed that the manuscript has been blinded to follow the double-blind peer review model, and approved the final version of the manuscript.
