# Supplementary material for: Coronary artery aneurysm formation after paclitaxel-coated balloon-only intervention for de novo coronary chronic total occlusion
Source: Front Cardiovasc Med. 2023 Jan 4;9:1039316. doi: 10.3389/fcvm.2022.1039316 (PMC9845697; doi:10.3389/fcvm.2022.1039316)
Supplement: Supplementary file 1 [file Data_Sheet_1.docx]

**SUPPLEMENTARY TABLE 1** ┃**Cochran–Armitage Test for Trend to Assess for the Presence of Association between the Severity of Coronary Dissection and Proportion of CAA.**

|  | Dissection type after DCB treatment | | | |
| --- | --- | --- | --- | --- |
|  | None | A | B | C |
| CAA | 0 | 0 | 3 | 4 |
| Non-CAA | 41 | 12 | 23 | 5 |

**SUPPLEMENTARY TABLE 2** ┃**Clinical Outcomes According to the Presence of Aneurysm after DCB-Only Treatment**

|  | CAA  N = 7 patients | Without CAA  N = 75 patients |
| --- | --- | --- |
| MACE | 0 | 4 (5.3) |
| All death | 0 | 2 (2.7) |
| Cardiac death | 0 | 2 (2.7) |
| Non-fatal myocardial infarction | 0 | 3 (4.0) |
| Target lesion revascularization | 0 | 1 (1.3) |
| Target vessel revascularization | 0 | 1 (1.3) |
| Target vessel thrombosis | 0 | 0 |
| Stroke | 0 | 1 (1.3) |
| Clinical follow-up duration, day, median (IQR) | 475 (393-1325) | 330 (1-981.5) |

Value is number (percentage).

Abbreviations: CAA = coronary artery aneurysm; MACE = major adverse cardiac events composed with cardiac death, non-fatal myocardial infarction, target vessel revascularization, and target vessel thrombosis; IQR = interquartile range
